# Abnormal Drop Formation from Copper Films via Detachment

**DOI:** 10.3390/ma18225169

**Published:** 2025-11-13

**Authors:** Heng-Zhi Liu, Xue-Qi Lv, Xiong-Ying Li

**Affiliations:** 1 School of Materials Engineering, Shanghai University of Engineering Science, Shanghai 201620, China; hz_liu_2025@163.com (H.-Z.L.); xqlyu@sues.edu.cn (X.-Q.L.); 2 Key Laboratory for Liquid-Solid Structural Evolution and Processing of Materials, Ministry of Education, Shandong University, Jinan 250061, China

**Keywords:** copper liquid films, drop formation, pinch-off, coalescence, carbon substrates, molecular dynamics simulations

## Abstract

Contacted liquid fluids, in most cases, have the tendency to directly merge into a single larger fluid to minimize the surface energy. We present an abnormal drop formation process of contacted Cu liquid films with a radius of 101.7 Å or larger on carbon substrates by using molecular dynamics simulations. The formation process consists of consecutive pinch-off and full coalescence stages connected by detachment. The dominant motions of the bridge, away from the center and downward to the substrate, lead to the pinch-off of the initially connected droplets. The motions of the droplets, which are near each other at all times, leads to the repeated contact and full coalescence of the separated droplets. The abnormality is attributed to the competition between the motions of the droplets and the tiny liquid bridge that connects the droplets. The influence of the surface structures of substrates, especially carbon nanotubes, on the formation behavior is discussed by analyzing the mean square displacement, velocity fields, and density and scaling profiles. This study provides guidance for controlling drop formation behavior by regulating the surface structures of carbon substrates.

## 1. Introduction

Drop formation has gained considerable attention not only because it plays a central role in spray printing [1,2], emulsion formation and colloidal dispersions [3,4] but also due to the richness of the underlying physics. First, breakup and coalescence during drop formation are finite time singular events during which the liquid topology changes from a single drop or thread into two or more drops or vice versa [5,6]. Second, near the singularity, right before pinch-off or just after coalescence, the dynamic of the tiny bridge that connects two drops exhibits temporally self-similar behavior, i.e.,; the shape near the singularity changes in time only by a change in scale [7,8,9]. Third, the self-similar behavior of varying viscosity drops is initial-condition independent and typically follows three regimes [10], in which the capillary force following the Laplace equation balances with inertia and viscosity [11,12,13]. The dominated regimes can be simply estimated by the dimensionless Reynolds number.

Research concerning drop formation has mainly focused on two aspects. On the one hand, much research has been performed on impacting [14], coalescence dynamics [15,16], pinch-off [17] and detachment dynamics [18,19,20]. Since the pioneering study on the self-similar recoil of a liquid sheet upon its rupture by Keller and Miksis [21], most research has focused on the singularity near the breakup and coalescence points for varying-viscosity drops [11,22]. In terms of high-viscosity drops, most of the dynamics are described by a fully viscous regime, where inertial forces are neglected and the bridge size is characterized as time *t* [10,17]. According to the widely accepted Reynolds number, the dynamic of low-viscosity liquids such as water is expected to follow the inertial regime. However, many scholars have predicted that viscous forces would not give way to the inertial ones until a certain time after contact, as indicated by the bridge size, which gives a growth switching from *t* to *t*^1/2^ for coalescence [17,22] and to *t*^2/3^ for pinch-off [10,11]. The difference of exponents between formation and vanishment is attributed to the complications introduced by the substrate and the contact angle according to the result from Eddi et al. [9], who conducted a qualitative study on the significance of substrates in drop formation. On the other hand, particular attention has been focused on the detachment of metallic films during drop formation on hydrophobic surfaces, which is inspired by the strategies of the amazing climbing ability of geckos [23,24] and the self-cleaning effects of lotus leaves [25,26]. Numerous experimental and theoretical investigations have confirmed that the continuous metallic films contract far from equilibrium due to the large surface energy [27], and they are unstable with respect to forming discrete nanodroplets [28] and to the detachment from substrates driven by inertial forces [29,30]. The above-mentioned findings have verified the notion that drop formation is a singular event, providing the possibility to understand the mechanism of drop formation from the perspective of a single stage, i.e., pinch-off (PO), full coalescence (FC), or detachment.

However, PO, detachment, and FC stages occur not individually but rather alongside or in a successive process in practice. Moreover, the drop in the merging process occasionally defies surface tension by PO prior to the occurrence of FC, which had been confirmed by the results from Blanchette and Bigioni [17], who demonstrated that merging does not always proceed to completion when a stationary drop coalesces with an underlying reservoir of identical fluid. Therefore, we are concerned about whether there exists one case in which PO, detachment, and FC occur successively and how the surface structure of substrates influences the deformation behavior. In this work, we carry out molecular dynamics simulations of Cu liquid films that are just coming into contact on three kinds of carbon substrates (CSs) to reveal the abnormal drop formation process which follows PO and FC stages connected by the detachment and the control of the abnormality by regulating the surface structure of the substrate.

## 2. Methods

The initially simulated systems are obtained by three steps. Firstly, the Cu films in circular shapes and a thickness of 15 Å but with different radii (*R*) ranging from 54.24 to 155.94 Å are extracted from a bulk liquid Cu at 1500 K. The extracted films are then deposited at a distance of 3.225 Å above the CSs with the connecting line of the center of each film parallel to the CSs. Herein, we selected three types of CSs due to the dewettability of Cu droplets on CSs, because the dewettability plays an important role in the contraction and detachment of liquid droplets on substrates. One is the double-wall graphene (DG). The other two are vertically and horizontally placed (10, 10) carbon nanotubes (VCNTs and HCNTs) arranging at monolayers and with an interval distance of 3.4 Å between the two adjacent nanotubes. Although all the contact angles of a Cu droplet on these CSs are approximately 130°, the different surface structures between the DG and CNTs and the different orientations between the VCNTs and the HCNTs would lead to different motions of the liquid bridges and the Cu droplets. All the substrates are placed in the *xoy* plane with the half-top parts fixed at the same temperature as the Cu films and the remaining parts kept fixed. Finally, every Cu–C system is immersed in a simulation box with a dimension of 700 × 700 × 50 Å^3^. At each side of the *x* and *y* directions, the length of each substrate is 20 Å larger than the Cu films to avoid any edge effect. Periodic boundary conditions are applied in the *x* and *y* directions.

MD simulations are carried out on the Cu–C systems under the constant-volume and constant-temperature (NVT) ensemble by means of the large-scale atomic/molecular massively parallel simulator (LAMMPS) package in the version of ‘LAMMPS-64bit-9Oct2020’ provided on https://rpm.lammps.org/windows/legacy/64bit/index.html (accessed on 10 November 2025) [31,32]. The interactions among Cu atoms are described by an embedded atom method (EAM) potential [33]. The C-C interaction is modeled by an adaptive intermolecular reactive empirical bond order (AIREBO) potential [34]. Due to the fact that metal and carbon can only form soft bonds via charge transfer from the π electrons in the *sp2* hybridized carbon to the empty 4 s states of metal [35,36,37], we utilize the 12–6 Lennard–Jones (LJ) potential with a well depth *ε* = 0.01 eV and size parameter *σ* = 3.225 Å to describe the Cu–C interaction [29,38]. This LJ potential was found to reproduce the equilibrium contact angle as 133° for Cu on pristine graphene. These values are in good agreement with the experimental one 140° [39]. The temperature is controlled by the Nose−Hoover method, and the time integration of the Newton’s equation of motion is undertaken using the velocity Verlet algorithm [40] with a time step of 1.0 fs. All the MD simulations are run for 500 ps to ensure the FC of Cu films. The dynamics snapshots of the drop formation process are obtained by the software OVITO in the version 3.5.1 provided on https://www.ovito.org/ [41].

The instantaneous self-diffusion coefficient of the Cu atom, *D_j_* (*j* = *x*, *y* and *z*), is calculated by the method based on the Einstein diffusion law:

$$D=\underset{t\to \infty }{\lim}\frac{\langle {\left|r_{i}(t)-r_{i}(0)\right|}^{2}\rangle }{6t}$$

where *ri(t)* is the position of the atom *i* at the time *t*, while 〈⋅⋅⋅〉 denotes an average over all water molecules.

The instantaneously mean-squared displacement (MSD) of Cu atoms is calculated, including all effects due to atoms passing through periodic boundaries, as indicated by the following function:

$$MSD(\delta t)=\left(\frac{\left|\overset{⃑}{r}(2)\right.-{\left.\overset{⃑}{r}(1)\right|}^{2}+\left|\overset{⃑}{r}(3)\right.-{\left.\overset{⃑}{r}(2)\right|}^{2}+\ldots +\left|\overset{⃑}{r}(n/2+1)\right.-{\left.\overset{⃑}{r}(n/2)\right|}^{2}}{n/2}\right)/N$$

where δt = 1 ps, *n* = 1000 time steps, and *N* is the total number of Cu atoms; then, the *D_j_* is derived from the MSD-*t* curve.

## 3. Results and Discussion

Simulations were carried out on the Cu–DG system to reveal the dynamics and mechanism of the complicated drop formation; then, they were carried out on the Cu–VCNT and Cu–HCNT systems to analyze the effect of the surface structure of the substrate on the formation behavior of the Cu drops.

### 3.1. Drop Formation on DG

**Figure 1:**
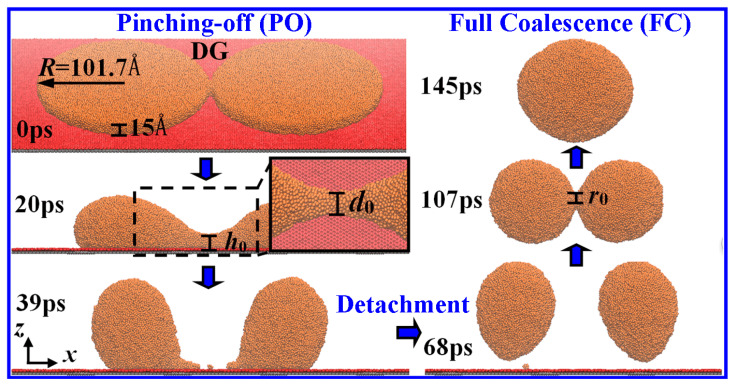
Snapshots of the drop formation via the contractions of two just-contact liquid Cu films on the DG. Both the films are in the thickness of 15 Å and the radii of *R* = 101.7 Å. *h*_0_, *d*_0_ and *r*_0_ denote the size of the tiny bridge that connects drops. The initial bridge pinches off at 39 ps.

Figure 1 shows snapshots of the unexpectedly complex drop formation via the contractions of two liquid Cu films (*R* = 101.7 Å) on the DG surface (see Video S1 in the Supporting Information). The films, once in contact, form a thin liquid bridge which rapidly widens, which is accompanied by capillary waves emitting to race up the film edge. If the growth of the bridge continues, the merging Cu films will be expected to further coalesce into a single large drop, which is inspired by the strategy of reducing the surface area to decrease the surface energy. On the contrary, the bridge narrows inward after a certain period and pinches at 39 ps as a result of the fast contraction of each film. The contracted films deform into two separate drops, which unexpectedly detach from the substrate at 64 ps due to the dewettability [27,28] and meanwhile gradually approach each other. The approaching drops contact at 107 ps and fully coalesce at 145 ps, resulting in the formation of a larger drop.

**Figure 2:**
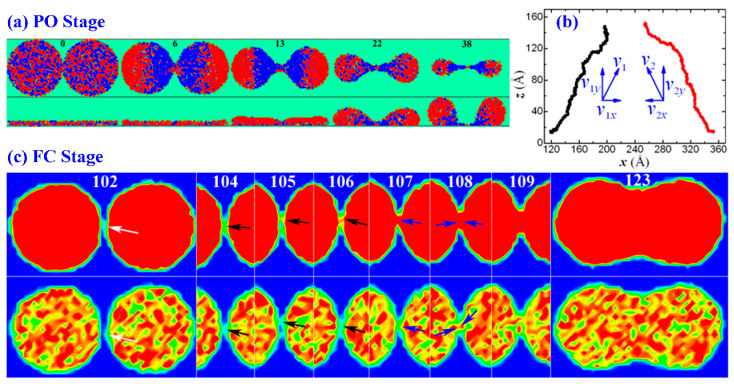
(**a**) The top and side views of velocity fields of Cu films on DG at *t* = 0, 6, 13, 22 and 38 ps. Both the films have a thickness of 15 Å and radii of *R* = 101.7 Å. The velocities with red and blue indicate the motions toward and away from the center of the bridge in the top view and the motions upwards and downwards in the side view, respectively. (**b**) The trajectories of the mass center of the contracting drops in the *xoz* plane indicate that the drops have the tendency to approach each other during the entire formation process. (**c**) The time evolution of density profiles (the top row) and coordination (the bottom row) at *t* = 102, 104, 105, 106, 107, 108, 109 and 123 ps. The surfaces of Cu drops break up at *t* = 104–106 ps, marked by the black arrows. The newly formed bridge grows up at *t* > 106 ps, labeled by blue arrows.

To gain insight into the mechanism behind the complexity of the drop formation, we analyze the motions of the bridge and Cu drops. Figure 2a shows the time-evolution velocity fields of the bridge and Cu drops along the *x* and *z* directions. With respect to the bridge, the motion along the *x* direction (top view) shows that the number of Cu atoms that move toward the center (red) is much smaller than the number that move away from the center (blue), and these further decrease as the process proceeds. This suggests that the motion away from the center plays a dominant role. Moreover, the motion along the *z* direction (side view) shows that the bridge has the tendency to vanish due to the dominant role of the downward motion (blue) in this direction. Due to the combined effect of the dominant motions in these two directions, the bridge disappears faster than it grows after a certain timescale, resulting in the PO. In terms of each drop, it mainly contracts to its center at the beginning, i.e., from 0 to 13 ps, but the tendency of each drop to move to the bridge becomes more obvious after 13 ps due to the increase in the number of the atoms that move to the bridge (red). A better understanding of this trend can be gained by the trajectory profiles of the two drops in the *xoy* plane, as shown in Figure 2b. Clearly, every drop gradually and consistently approaches the other drops before the FC stage. The velocity component in the *x* direction gives rise to the contact and the FC of Cu drops, and the velocity component in the *z* direction of each Cu drop leads to its detachment. That is to say, though the vanishment of the bridge occurs, the separating drops tend to approach each other, paving the way for the FC stage. Figure 2c presents the time-evolution density profiles (the top row) and coordination distribution (the bottom row) in order to illustrate the FC stage and the dynamic of the liquid–liquid interface. The FC of Cu drops undergoes three steps. Firstly, near the coalescing region, the surface of the right drop firstly becomes protuberant, which is denoted by white arrows in the first snapshot (*t* = 102 ps) at the top row. This step is quite similar to the gas drainage in the liquid coalescence in real space. Secondly, the surfaces of Cu drops break up at *t* = 104–106 ps (marked by the black arrows), resulting in the formation of a new liquid bridge. Thirdly, the newly formed bridge grows up at *t* > 106 ps (labeled by blue arrows) until the FC of the Cu drops. The fluctuations on the drop surface indicate that the liquid–liquid interface emits capillary waves to the drop surfaces, and this is of significance in the spontaneous process of droplet coalescence [42,43].

**Figure 3:**
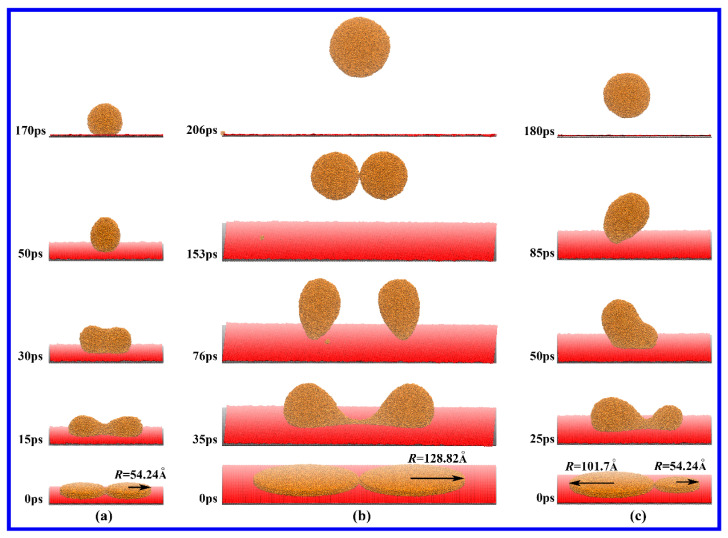
Snapshots of the drop formation from two liquid Cu films that have just come into contact on DG. All the films are in the thickness of 15 Å. The radius (*R*) of the film is labeled by the black arrow. (**a**) *R* = 54.24 Å for both films, (**b**) *R* = 128.82 Å for both films, (**c**) *R* = 54.24 Å for one film and *R* = 101.7 Å for the other film.

Do all of the liquid Cu films with a thickness of 15 Å and an any value of radii display the above-mentioned abnormal drop formation process on DG? The answer is “NO”. If the *R* is smaller than 101.7 Å, such as *R* = 54.24 Å, the bridge that connects the two films gradually will grow up until the FC of the Cu drops without the PO of the bridge and the detachments of Cu drops (Figure 3a). Conversely, if the *R* is larger than 101.7 Å, such as *R* = 128.82 Å, a similar drop formation process with the case of *R* = 101.7 Å will occur during the contractions of Cu films (Figure 3b). In addition, we found that if one film has the radii *R* = 54.24 Å and the other film has the radii *R* = 101.7 Å, the smaller Cu drop will be dragged by the larger one via the bridge to detach from the DG substrate (Figure 3c). In this case, the PO of the bridge, however, is not observed. Based on these findings, we can reason that the occurrence of the PO should satisfy two conditions. One is that the *R* values of both films are identical. The other one is that the *R* values are large enough to provide enough surface energy to ensure that the contractions of the films are faster than the growth of the bridge. In addition, the deceased surface energy, attributed to the contractions of Cu films, provides a large velocity component in the *z* direction so that the Cu drops can overcome the force from the substrate to pinch off.

**Figure 4:**
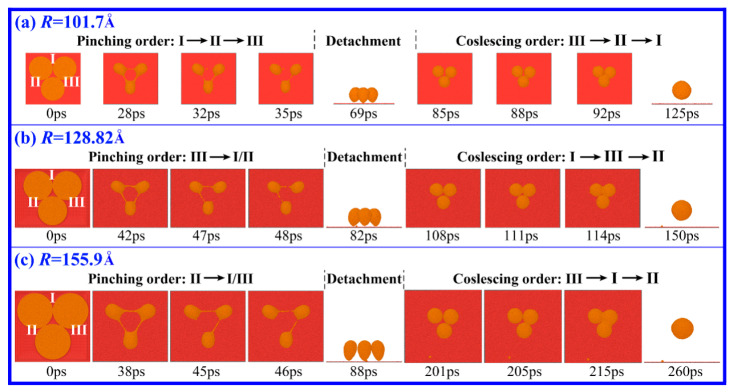
Snapshots of the drop formation from three liquid Cu films that have just come into contact on DG. Cu films are in a thickness of 15 Å but different radii: (**a**) *R* = 101.7 Å, (**b**) *R* = 128.82 Å and (**c**) *R* = 155.9 Å. The bridges connecting films or droplets are numbered as I, II and III.

If the number of liquid Cu films increases from two to three, what will happen in the motions of the bridges and Cu drops? To reveal the deformation behavior of three liquid Cu films with the same *R* that have just come into contact on DG, the cases of *R* = 101.7, 128.83 and 155.94 Å are studied. Comparing the deformation of three films with that of two films, there is one similar point and one different point. The similar point is that the drop formation undergoes the abnormal drop formation process: the initial generation of the tiny liquid bridge, the subsequent PO of the bridge followed by the detachment of drops, and the final FC of all drops (Figure 4). The consistency provides support for the possibility of a connection between the PO and FC stages by detachment. The different point is that the deformation of three films is more complicated. At *R* = 101.7 Å, the bridges pinch in the following order: I, II and III at the PO stage, but they reform in the reverse order: III, II and I at the FC stage (Figure 4a). At *R* = 128.82 Å, the order of the PO is III to I/II, and the order of the FC is I, III and II. At *R* = 155.94 Å, the order of the PO is II to I/III and the order of the FC is III, I and II. These findings make it seem like the orders of PO and FC in the case of the three films are random. In addition, we found that if the interval distances of the liquid films are larger than 3 Å, the films only contract to form separate drops without the formation of the tiny bridge and the above-mentioned abnormal drop formation process. This means that the generation of the tiny liquid bridge plays an important role in the anomalies, but it does not play a dominant role. As shown in Figure 3a, the films rapidly contact and merge into a larger drop without PO or even detachment regardless of the generation of a liquid bridge.

### 3.2. Drop Formation on CNTs

**Figure 5:**
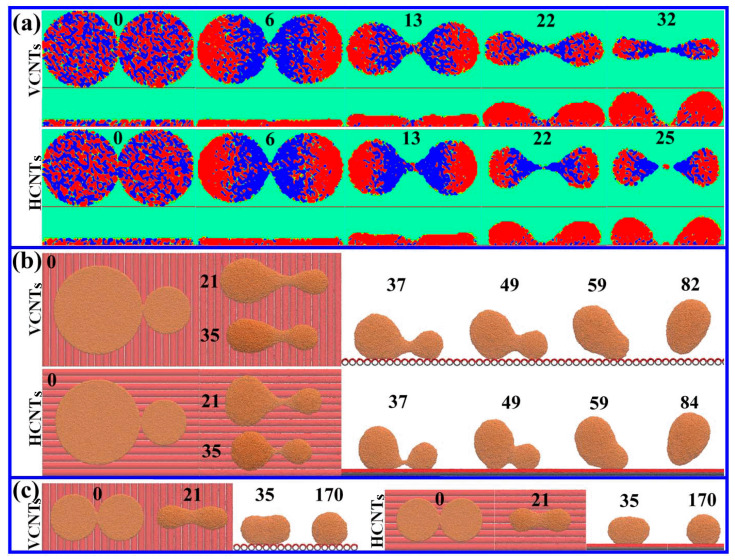
Snapshots of the drop formation via the contractions of two liquid Cu films that have just come into contact on CNTs. (**a**) The top and side views of the velocity fields of Cu films (*R* = 101.7 Å) at *t* = 0, 6, 13, 22 and 25 (32) ps. The PO occurs at 32 ps on VCNTs and at 25 ps on HCNTs, indicating that PO is easier on CNTs than on DG. The velocities with red and blue indicate the motions toward and away from the center of the bridge in the top view and the motions upwards and downwards in the side view, respectively. (**b**) *R* = 101.7 Å for the left Cu film and *R* = 54.24 Å for the right Cu film. The coalesced drop detaches at 82 ps on VCNTs and at 84 ps on HCNTs. (**c**) *R* = 54.24 Å.

With respect to two Cu films, the similar abnormal drop formation on CNTs to that on DG is verified from Figure 5. In the cases of *R* ≥ 101.7 Å, the drop formation process follows the PO and FC stages connected by the detachment (Figure 5a). In the case of *R* = 54.24 Å for one film and *R* = 101.7 Å for the other film, the smaller drop that does not detach from the substrate gradually merges into the larger one and is dragged by the larger drop via the connecting bridge to detach from the substrate (Figure 5b). The PO of the bridge is not observed. In the case of *R* = 54.24 Å, the two Cu films contract and directly merge into a single drop without the PO and detachment (Figure 5c).

The differences between drop formation behaviors on CNTs and DG reflect the motions of the bridge and Cu drops. Figure 5a shows that the dynamic at the PO stage is quite different from that on DG. It can be seen that the Cu atoms, especially in the case of HCNTs, exhibit strongly upward motions (marked in red) in each drop but downward motions (marked in blue) in the bridge according to the side views of the motions in the *z* direction, thus leading to the earlier PO on CNTs (32 ps on VCNTs and 25 ps on HCNTs) than on DG (39 ps). Importantly, the films gradually change into relatively long and thin drops on VCNTs and into short and wide drops on HCNTs with respect to the top views of the motions along the *x* axis, signifying that the velocities along the *x* and *y* directions are quite different on CNTs. Such a phenomenon is also found in the case with one of the Cu drops in a smaller diameter such as *R* = 54.24 Å, as shown in Figure 5b. The liquid flow on VCNTs is elongated along the *x* axis due to the faster contracting speed in the *y* direction than in the *x* direction. Following an inverse contracting speed, the Cu drops on HCNTs deform into circular-like shapes. This consistency provides evidence for orienting the contraction of the liquid flow along different directions. Moreover, the detachment of the larger drop occurs prior to the FC, and this is opposite to that on DG, implying the importance of the surface structure on the pinching behavior. The effect, however, becomes weak with the radius of films decreasing to 54.24 Å (Figure 5c). All of the drops on different CNTs display nearly the same shape. This indicates that the effect of the surface structure on the pinching behavior becomes weaker and weaker due to the decrease in the size of the film. Therefore, the above-mentioned differences reveal that the controls of drop formation and the shape of the fluid during the formation process could be realized by tuning the surface structure of the substrate or sizes of the Cu films.

**Figure 6:**
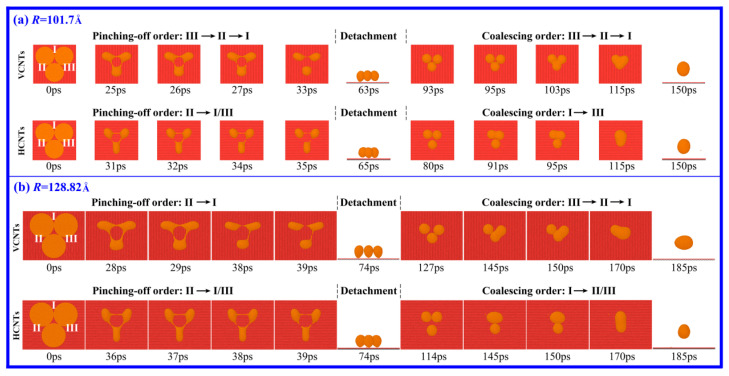
Snapshots of the drop formation from three liquid Cu films that have just come into contact on VCNTs and HCNTs. The Cu films have a thickness of 15 Å and radii of (**a**) *R* = 101.7 Å and (**b**) *R* = 128.82 Å. The bridges connecting films or droplets are numbered as I, II and III.

Figure 6 shows the snapshots of the deformation process of drops from the three tangent liquid Cu films on VCNTs and HCNTs. All Cu films have a thickness of 15 Å and a radius of *R* = 101.7 or 128.82 Å. As in the cases of two Cu films with *R* ≥ 101.7 Å, the formation process of the Cu drops also includes the PO and FC stages connected by detachment (see Video S2 in the Supporting Information). Differing from the cases of three Cu films with *R* ≥ 101.7 Å on DG, the vanishing of the tiny liquid bridges at the PO stage and the formations of the liquid bridges at the FC stage seem to be controllable. At the PO stage in the cases of *R* = 101.7 Å (Figure 6a), the top two drops on VCNTs and the bottom drop on HCNTs are obviously elongated along the vertical direction to the axes of the CNTs, indicating that the contraction of the liquid film is faster in the direction parallel to the axes of the CNTs than in the direction vertical to the axes of the CNTs. Thus, at the PO stage, liquid bridges II or III (or both) vanish before bridge I on both the VCNTs and HCNTs; and at the FC stage, the liquid bridges form in the identical order on VCNTs but in the reverse order on HCNTs. Similar phenomena are observed in the cases of *R* = 128.82 Å (Figure 6b). Therefore, it may be predicted that the control of both the shape of the drops and the dynamics of the bridge at either the PO or FC stage is available by appropriately selecting the substrate.

**Figure 7:**
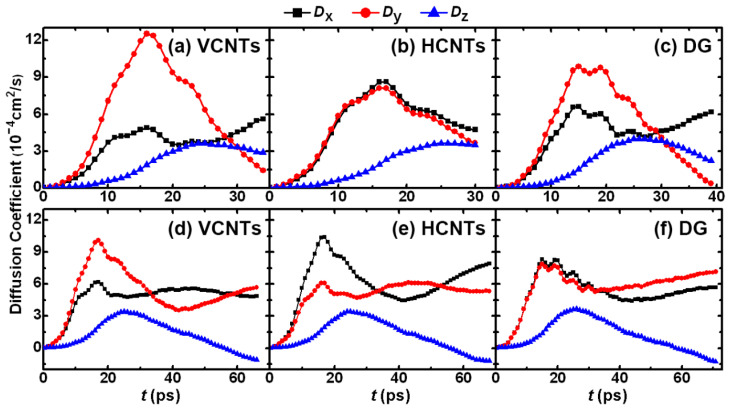
Diffusion coefficient (*D*) of Cu atoms along different directions at the PO stage on different substrates. (**a**–**c**) The *D* for the case of two films with *R* = 101.7 Å. (**d**–**f**) The *D* for the case of three films with *R* = 101.7 Å. The largest value of *D_x_* (*D_y_*) on HCNT (VCNT) indicates that the orientation of the drop formation along *x* (*y*) is enhanced on HCNT (VCNT).

To reveal the motions of Cu atoms along different directions at the PO stage, further research is carried out on the time-evolution diffusion coefficient (*D*) of the Cu atoms. Figure 7a–c show the *D* in the cases of two films with *R* = 101.7 Å. On the flat surface (DG), the *D_y_* is slightly larger than the *D_x_*. However, on VCNTs, the *D_y_* significantly increases and displays the highest peak value, but the *D_x_* greatly decreases and displays the lowest peak value. On HCNTs, the *D_x_* (*D_y_*) slightly increases (decreases) and displays the largest peak value. Moreover, *D_x_* is nearly equal to *D_y_*. That is to say, the contraction of films along the *y* direction is slightly faster than that along the *x* direction on DG, and it is further enhanced on VCNTs, while the contraction along the *x* direction is significantly restrained on VCNTs but enhanced on HCNTs, which suggests that the *D* is quite sensitive to the surface structure of the substrate except for the *D_z_*, which is nearly the same on different substrates. Figure 7d–f give the *D* values for the cases of three films with *R* = 101.7 Å. The *D_z_* is independent of the surface structure, and it is in good agreement with that in the case of two films. However, the two lines *D_x_*/*D_y_*-*t* nearly overlap on DG, especially at the beginning. On VCNTs, *D_y_* significantly increases but *D_x_* gradually approaches a constant value, which is in contrast to the behavior on HCNTs, where the contraction along the *x* direction is enhanced. Therefore, the analysis from Figure 5 confirms that the orientation of the drop formation can be effectively controlled by regulating the substrate, which may shed new light on the growth and coalescence of nanostructures [44].

**Figure 8:**
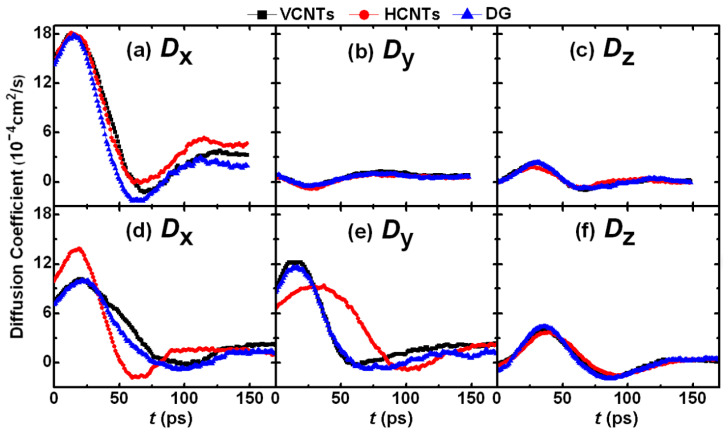
Diffusion coefficient (*D*) of Cu atom along different directions at the FC stage on different substrates. The time when the separated drops contact is set as *t* = 0 ps. (**a**–**c**) The *D* for the case of two films with *R* = 101.7 Å. The diffusion along the *x* direction plays a dominant role in drop formation due to the far larger value of the *D_x_* compared with the others. (**d**–**f**) The *D* for the case of three films with *R* = 101.7 Å. The diffusion in the *y* direction is as important as that in the *x* direction due to the nearly equal values of the *D_x_* and *D_y_*, which are much larger than the *D_z_*.

Figure 8 shows the *D* at the FC stage for the cases of two and three films with *R* = 101.7 Å. In terms of the cases with two films, Figure 8a–c reveal that the diffusion along the *x* direction plays a dominant role in the FC due to the *D*_x_ value being much larger than the *D*_y_ and *D*_z_ values. Moreover, the *D*_y_/*D*_x_-*t* curves nearly overlap with respect to different substrates. However, in the cases of three films, both the diffusions along the *x* and *y* directions play important roles in coalescence due to the fact that both the *D*_x_ and *D*_y_ are far larger than the *D*_z_, as indicated by Figure 8d–f. Notably, the overlaps of the *D*-*t* lines suggest that the *D* on VCNTs is generally equal to that on DG. However, on HCNTs, the *D*_x_ varies rapidly and then reaches the plateau at about 85 ps, while the *D*_y_ changes gradually and becomes constant after 150 ps. This means that the diffusions along the *x* and *y* directions on this substrate approach the equilibrium at different times, which is possibly because the drops on HCNTs are greatly elongated in the *y* direction (Figure 4), finally leading to the later equilibrium in the *y* direction compared with the *x* direction.

**Figure 9:**
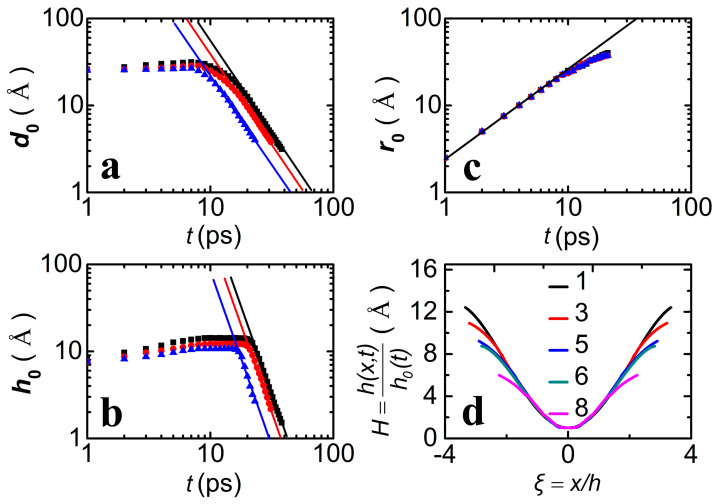
Scaling profiles of the bridge at the pinching and coalescing points for the case of two films with *R* = 101.7 Å. (**a**–**c**) The minimum width (*d*_0_), height (*h*_0_) and radius (*r*_0_) of the tiny bridge in the cases DG (squares), VCNT (circles) and HCNT (triangles), scaling with ~*t*^2^, ~*t*^3^ and ~*t*^2/3^, respectively. The high value of the exponent of the first two scales illustrates the critical role of inertial force in the dynamics of drop formation at high temperature. The overlap of the *r*_0_-*t* curves implies the independence of the substrate on the self-similarity at the FC stage. The solid lines are fitting curves according to the corresponding scales. (**d**) Rescaled profiles $H=h(x,t)/h_{0}(t)$ for 5 different times *t* = 1, 3, 5, 6 and 8 ps after contact during the FC stage.

Figure 9 shows the sizes of the tiny bridge as a function of time, including the minimum width (*d*_0_), height (*h*_0_) and radius (*r*_0_). *d*_0_ and *h*_0_ show a steep decline before the pinching point, while *r*_0_ displays a linear increase, but all these sizes are proportional to *t*^k^, characterized as *d*_0_~*t*^2^, *h*_0_~*t*^3^ and *r*_0_~*t*, which indicates that the dynamic of the bridge exhibits self-similarity at both the PO and FC stages. Compared to *d*_0_, *h*_0_ displays a sharp decrease. The overlap of the *r*_0_-*t* curves for different systems and the same scale *r*_0_~*t* indicate that the self-similarity at the FC stage is independent of the substrates. In addition, the rescaled profiles reveal that the self-similarity at the FC stage is remarkable within 10 ps and becomes weak as the drops coalesce. However, the exponent of each stage for the Cu droplet is much larger than that for the ambitious spherical drop scaling with *h*_0_~*t*^2/3^ at the PO stage and *h*_0_~*t*^1/2^ at the FC stage [10,11], which implies that the formation of the high-temperature liquid drop exhibits a quite different dynamic.

**Figure 10:**
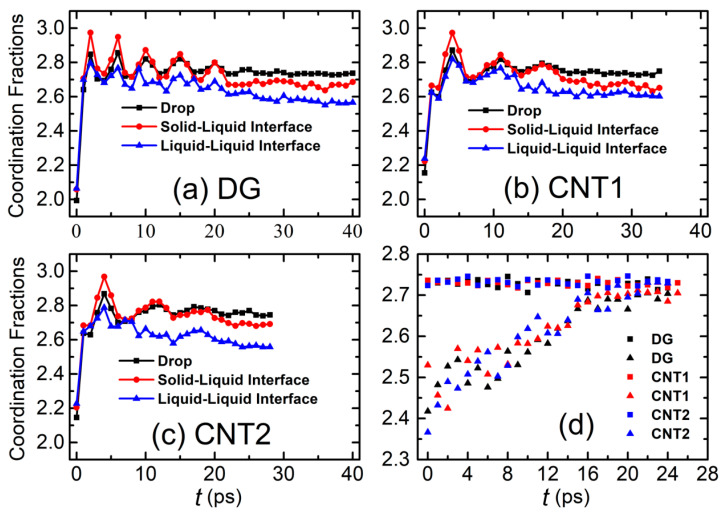
Coordination fractions of drops (squares), the solid–liquid interface (circles) and liquid–liquid interface (triangles) versus time for the cases of two films with *R* = 101.7 Å. (**a**–**c**) At the PO stage, the fluctuations in the curves at the beginning imply the weak stability of the initial coalescence. (**d**) At the FC stage, the steady increase in coordination fractions of the liquid–liquid interface reveals the growth of the bridge.

To explore the instantaneously local and entire structure of the fluid, the coordination fractions as functions of time are plotted in Figure 10. During the PO stage, all of the coordination fractions show dramatic fluctuations at the beginning, but they gradually become stable and decrease gradually before the pinching point, implying the weak stability of the initial coalescence and the tendency of PO. It is noted that the liquid–liquid interface shows the lowest coordination fraction especially during the time when the liquid bridge narrows inward, which is followed by the solid–liquid interface, suggesting that the tiny bridge is in a stretching state, which provides further evidence for the vanishment of the bridge. As for the FC event, though the coordination fractions of the liquid–liquid interface are much smaller than those of the drops, they increase steadily and finally become equal to those of the drops, implying the steady growth of the bridge and the coalescence of drops at this stage.

## 4. Conclusions

In summary, MD simulation has been carried out to study the unexpected drop formation of contacted thin films and reveal the possibility of connecting PO and FC by the detachment resulting from the dewettability. The complex process is mainly attributed to the combined competitions between the motions of bridges and drops along the *x* and *z* directions. Further research on the Cu–CNT system shows that the anomalies of the drop formation are quite sensitive to the surface structure of the substrate due to the differences in the diffusions along the *x* and *y* directions on different substrates. Moreover, the evolution of the shapes near the pinching and coalescing points implies the self-similarity of the bridge that connects these two drops, which is characterized as *d*_0_/*h*_0_/*r*_0_~*t*^k^. The exponent *k* is much larger than that of the ambitious low-viscosity drops. In the cases of three Cu films, the pinching-off and coalescing orders are random on DG but depend on the surface structure of the substrate on CNTs. The anomalies in the Cu drop formation and the controlled orientation of the formation not only shed light on the formation dynamics of metallic liquid nanofilms but also pave the way for new and promising applications of metallic drops in nanofluidic devices and 3D printing.

## Data Availability

The original contributions presented in this study are included in the article/Supplementary Material. Further inquiries can be directed to the corresponding author.

## Supplementary Materials

The following supporting information can be downloaded at https://www.mdpi.com/article/10.3390/ma18225169/s1, Video S1: Dynamics of the drop formation from two Cu films with the radius *R* = 101.7 Å on DG. Video S2: Dynamics of the drop formation from three tangent Cu films with *R* = 101.7 Å on VCNTs.
